# Androgen receptor gene status in plasma DNA associates with worse outcome on enzalutamide or abiraterone for castration-resistant prostate cancer: a multi-institution correlative biomarker study

**DOI:** 10.1093/annonc/mdx155

**Published:** 2017-05-03

**Authors:** V. Conteduca, D. Wetterskog, M. T. A. Sharabiani, E. Grande, M. P. Fernandez-Perez, A. Jayaram, S. Salvi, D. Castellano, A. Romanel, C. Lolli, V. Casadio, G. Gurioli, D. Amadori, A. Font, S. Vazquez-Estevez, A. González del Alba, B. Mellado, O. Fernandez-Calvo, M. J. Méndez-Vidal, M. A. Climent, I. Duran, E. Gallardo, A. Rodriguez, C. Santander, M. I. Sáez, J. Puente, D. Gasi Tandefelt, A. Wingate, D. Dearnaley, PREMIERE Collaborators, Spanish Oncology Genitourinary Group, F. Demichelis, U. De Giorgi, E. Gonzalez-Billalabeitia, G. Attard

**Affiliations:** 1 Centre for Evolution and Cancer, The Institute of Cancer Research, London, UK;; 2 Department of Medical Oncology, Istituto Scientifico Romagnolo per lo Studio e la Cura dei Tumori (IRST) IRCCS, Meldola, Italy;; 3 Research Data Management and Statistics Unit, The Royal Marsden NHS Foundation Trust, London, UK;; 4 Department of Medical Oncology, Hospital Ramón y Cajal, Madrid;; 5 Department of Hematology & Medical Oncology, Hospital Universitario Morales Meseguer, IMIB-Universidad de Murcia, Murcia;; 6 Academic Urology Unit, The Royal Marsden NHS Foundation Trust, London, UK;; 7 Department of Medical Oncology, Hospital Universitario 12 de Octubre, Madrid, Spain;; 8 Centre for Integrative Biology, University of Trento, Trento, Italy;; 9 Oncology Unit, Institut Català d'Oncologia-Hospital Germans Trias i Pujol, Badalona;; 10 Department of Medical Oncology, H. Universitario Lucus Augusti, Lugo;; 11 Department of Medical Oncology, H.U. Son Espases Mallorca, Mallorca;; 12 Department of Medical Oncology, IDIBAPS Hospital Clinic, Barcelona;; 13 Department of Medical Oncology, Hospital de Orense, Orense;; 14 Department of Medical Oncology, Hospital Universitario Reina Sofía, Córdoba;; 15 Department of Medical Oncology, Instituto Valenciano de Oncología Valencia, Valencia;; 16 Department of Medical Oncology, Instituto de Biomedicina de Sevilla, IBiS/Hospital Universitario Virgen del Rocío/CSIC/Universidad de Sevilla, Sevilla;; 17 Department of Medical Oncology, H.U. Parc Taulí, Sabadell, Barcelona;; 18 Department of Medical Oncology, Hospital de León, León;; 19 Department of Medical Oncology, Hospital Universitario Miguel Servet, Zaragoza;; 20 Department of Medical Oncology, Hospital Regional y Hospital Virgen de la Victoria, Malaga;; 21 Department of Medical Oncology, Hospital Clínico San Carlos, Madrid, Spain;; 22 Division of Radiotherapy and Imaging, The Institute of Cancer Research, London, UK;; 23 Institute for Precision Medicine, Weill Cornell Medicine, New York, USA;; 24 Department of Medical Oncology, Universidad Católica San Antonio de Murcia-UCAM, Murcia, Spain

**Keywords:** castration-resistant prostate cancer, androgen receptor, plasma DNA, enzalutamide, abiraterone, biomarker

## Abstract

**Background:**

There is an urgent need to identify biomarkers to guide personalized therapy in castration-resistant prostate cancer (CRPC). We aimed to clinically qualify androgen receptor (*AR*) gene status measurement in plasma DNA using multiplex droplet digital PCR (ddPCR) in pre- and post-chemotherapy CRPC.

**Methods:**

We optimized ddPCR assays for *AR* copy number and mutations and retrospectively analyzed plasma DNA from patients recruited to one of the three biomarker protocols with prospectively collected clinical data. We evaluated associations between plasma *AR* and overall survival (OS) and progression-free survival (PFS) in 73 chemotherapy-naïve and 98 post-docetaxel CRPC patients treated with enzalutamide or abiraterone (Primary cohort) and 94 chemotherapy-naïve patients treated with enzalutamide (Secondary cohort; PREMIERE trial).

**Results:**

In the primary cohort, *AR* gain was observed in 10 (14%) chemotherapy-naïve and 33 (34%) post-docetaxel patients and associated with worse OS [hazard ratio (HR), 3.98; 95% CI 1.74–9.10; *P *<* *0.001 and HR 3.81; 95% CI 2.28–6.37; *P *<* *0.001, respectively], PFS (HR 2.18; 95% CI 1.08–4.39; *P *=* *0.03, and HR 1.95; 95% CI 1.23–3.11; *P *=* *0.01, respectively) and rate of PSA decline ≥50% [odds ratio (OR), 4.7; 95% CI 1.17–19.17; *P *=* *0.035 and OR, 5.0; 95% CI 1.70–14.91; *P *=* *0.003, respectively]. *AR* mutations [2105T>A (p.L702H) and 2632A>G (p.T878A)] were observed in eight (11%) post-docetaxel but no chemotherapy-naïve abiraterone-treated patients and were also associated with worse OS (HR 3.26; 95% CI 1.47–not reached; *P *=* *0.004). There was no interaction between *AR* and docetaxel status (*P *=* *0.83 for OS, *P *=* *0.99 for PFS). In the PREMIERE trial, 11 patients (12%) with *AR* gain had worse PSA-PFS (sPFS) (HR 4.33; 95% CI 1.94–9.68; *P *<* *0.001), radiographic-PFS (rPFS) (HR 8.06; 95% CI 3.26–19.93; *P *<* *0.001) and OS (HR 11.08; 95% CI 2.16–56.95; *P *=* *0.004). Plasma *AR* was an independent predictor of outcome on multivariable analyses in both cohorts.

**Conclusion:**

Plasma *AR* status assessment using ddPCR identifies CRPC with worse outcome to enzalutamide or abiraterone. Prospective evaluation of treatment decisions based on plasma *AR* is now required.

**Clinical Trial number:**

NCT02288936 (PREMIERE trial).

## Introduction

Inhibition of androgen receptor (AR) signaling with abiraterone or enzalutamide is now standard treatment at emergence of castration-resistant prostate cancer (CRPC). However, the duration of response is variable and overall survival (OS) in unselected patients is modest despite some patients having responses that last several years [1, 2]. There is therefore an urgent need to develop biomarker strategies to *a priori* identify CRPC patients who will derive minimal benefit from AR targeting and offer them an alternative treatment paradigm. Testing for plasma epidermal growth factor receptor (*EGFR*) mutations has FDA clearance for selection of mutant lung cancer patients for EGFR tyrosine kinase inhibitors and studies of plasma DNA in multiple indications have suggested clinical utility for monitoring of mutations or copy number (CN) gain [3–6]. 

Next-generation sequencing (NGS) and PCR-based studies have identified associations between *AR* CN gain detected in plasma and worse outcome with abiraterone or enzalutamide, in predominantly post-docetaxel CRPC cohorts [7–12]. *AR* gene aberrations are rare before hormone therapy but occur in metastases harvested at rapid warm autopsy from up to 60% of patients [13]. Using NGS on sequential plasma samples, we have identified two *AR* point mutations [2105T>A (p.L702H) and 2632A>G (p.T878A)] as associating with resistance to abiraterone, shown previously to be activated by prednisone or progesterone, respectively [7, 8, 14, 15]. For enzalutamide, the 2629T>C (p.F877L) point mutation has been reported as a resistance mechanism [16, 17] although a recent study suggested it is very uncommon [12]. Following a well-described roadmap for the implementation of a biomarker test into routine clinical practice [18], we aimed to optimize a droplet digital PCR (ddPCR) assay that is fit for purpose and can be widely implemented on plasma DNA in clinical laboratories. We sought to define *AR* CN and in a separate reaction, *AR* mutation status: 2105T>A and 2632A>G in patients considered for abiraterone and 2629T>C for patients treated with enzalutamide. We then aimed to obtain stage one biomarker clinical qualification for associations with clinical outcome on enzalutamide or abiraterone in chemotherapy-naïve and post-docetaxel CRPC patients treated in one of three biomarker protocols.

## Materials and methods

### Study design

This was a multi-institution analysis of plasma samples collected prospectively in studies with the primary aim of biomarker evaluation. The objectives were defined after sample collection but before plasma analysis. Our first objective was to determine the correlation between ddPCR testing for plasma *AR* and an orthogonal approach, NGS, in samples collected before starting treatment and after disease progression. Our second objective was to evaluate associations between pre-treatment plasma *AR* and clinical outcome in a primary cohort, representative of both pre- and post-docetaxel patients, and test for interactions with prior chemotherapy exposure. As no trial to date has randomized patients between first-line enzalutamide or abiraterone and taxanes, we combined data from four cohorts of men recruited to two biomarker protocols and defined by treatment with enzalutamide or abiraterone and prior chemotherapy status. Our third objective was to test our ddPCR assay in a second cohort of chemotherapy-naïve men treated with enzalutamide in the PREMIERE trial.

### Participants

The primary cohort included patients participating in one of two protocols separately approved by the Institutional Review Board of the Royal Marsden (RM), London, UK (REC 04/Q0801/6), and Istituto Scientifico Romagnolo per lo Studio e la Cura dei Tumori (IRST), Meldola, Italy (REC 2192/2013). Docetaxel in this cohort was only used in the CRPC setting. The second cohort was the PREMIERE trial (EudraCT: 2014-003192-28, NCT02288936) that was sponsored and conducted by the Spanish Genito-Urinary oncology Group (SOGUG). The trial was approved by the independent review board at each participating site. This trial was designed to analyze the predictive value of the gene fusion *TMPRSS2-ETS* in response to enzalutamide in patients with prostate cancer. Exploratory endpoints included circulating cell-free DNA and circulating tumor cell (CTC) analysis. Data emerging after the trial was designed and initiated [7, 19, 20] led the PREMIERE Trial Management Group to additionally prioritize two alternative biomarkers for evaluation, namely AR-V7 detected in CTCs as described previously [19] and plasma *AR*. *TMPRSS2-ETS* analyses are on-going and will be reported elsewhere. Preliminary AR-V7 data were presented in abstract form at the ESMO 2016 Annual Meeting [21] and will be published elsewhere. These analyses were based on the first censor cut-off, date May 2016. A second data analysis is planned at a predefined time-point when enough events have occurred to address the primary endpoint.

In both cohorts, patients were required to have histologically confirmed prostate adenocarcinoma without neuroendocrine differentiation, progressive disease despite ‘castration levels’ of serum testosterone (<50 ng/dl), on-going LHRH analogue treatment or prior surgical castration and no prior treatment with enzalutamide or abiraterone. Additional selection criteria by cohort are specified in the supplementary Appendix S1, available at *Annals of Oncology* online. The choice of therapy in the primary cohort was at the discretion of the treating physician, either enzalutamide 160 mg once a day or abiraterone 1 g once a day and prednisone 5 mg twice daily. In the PREMIERE trial, all patients received enzalutamide 160 mg once a day. Treatment in both cohorts was administered continuously until evidence of progressive disease or unacceptable toxicity. The studies were conducted in accordance with the Declaration of Helsinki and the Good Clinical Practice guidelines of the International Conference of Harmonization. Written informed consent was obtained from all patients.

### Procedures

Peripheral blood samples were collected within 30 days of treatment initiation and plasma aliquots stored at −80 °C. ddPCR assays were carried out as described in detail in supplementary Appendix S2, available at *Annals of Oncology* online. For each individual sample *AR* CN was estimated using each of the reference genes *NSUN3*, *ElF2C1*, and *AP3B1* and using *ZXDB* at Xp11.21 as a control gene to determine X chromosome CN. *AR* mutation detection assays were carried out for the *AR* mutations 2105T>A (p.L702H), 2632A>G (p.T878A), and 2629T>C (p.F877L) with a limit of detection of 1%–2% using an input of 2–4 ng of DNA. For NGS on plasma and patient-matched germline DNA, we used a customized AmpliSeq targeted gene panel including *AR*, sequenced on an Ion Torrent Personal Genome Machine or Proton as described previously [7, 8]. Computational analysis estimating the plasma DNA tumor content, *AR* CN quantitation and point mutation detection (with a sensitivity of 98%–99% depending on position and coverage) was carried out as previously [8].

Serum prostate-specific antigen (PSA) was assessed within 1 week of starting treatment and monthly thereafter. Radiographic disease was evaluated with the use of computed tomography and bone scan at the time of screening and every 12 weeks on treatment. In the primary cohort, serum lactate dehydrogenase (LDH) and alkaline phosphatase (ALP) were also measured within 1 week of starting treatment. In PREMIERE, CTCs were evaluated pre-treatment using the AdnaTest for Prostate Cancer (Qiagen GmbH, Germany) as described previously [21].

### Outcomes

For the primary cohort, the primary endpoint was OS. The secondary endpoints were progression-free survival (PFS) (biochemical and/or radiographic and/or clinical) and PSA response. For PREMIERE, the primary endpoint was PSA-PFS (sPFS). Secondary endpoints included radiographic-PFS (rPFS), OS and PSA response. OS was calculated from initiation of therapy to death from any cause. Patients still alive at time of last follow-up were censored. PFS was calculated from the first day of enzalutamide or abiraterone therapy to the date of progression disease or death. Radiographic progression was defined using Response Evaluation Criteria in Solid Tumors version 1.1. PSA decline was evaluated according to Prostate Cancer Working Group (PCWG2) guidelines [22].

### Statistical analyses

An R script [23] was developed to identify the optimal *AR* CN cut-point that associated with OS in the primary cohort, using maximum log-likelihood as correlative statistics in a multivariable Cox regression model by an approach described previously (supplementary Appendix S3, available at *Annals of Oncology* online) [24]. The process was bootstrapped with 30,000 iterations to provide the measures of dispersion. Remaining analyses were conducted using Stata/MP 13.1 for Windows. Time-to-event outcomes were evaluated using Kaplan–Meier survivor estimates, log-rank test and univariate and multivariable Cox-proportional hazards models. The association of clinically relevant baseline factors (previously showed to be associated with prognosis [25, 26]) with OS and PFS was examined using a univariate Cox regression model. A multivariable Cox regression model was then carried out with a stepwise procedure to identify the prognostic factors for OS and PFS with a significance level of <0.05 for entry into the model. All tests were two-sided and an *α*-error of 5% was considered as significant. Odds ratios of PSA response were determined using a 2×2 contingency table and significant differences using Fisher’s exact test (supplementary Appendix S3, available at *Annals of Oncology* online).

## Results

### Clinical characteristics of the primary cohort

In the primary cohort, we had 171 men who started treatment with enzalutamide or abiraterone between 31 January 2011 and 9 June 2016, 73 before docetaxel and 98 after. All had received bicalutamide. Patient and treatment characteristics at the time of sample collection are detailed in Table 1.

**Table 1:** Baseline characteristics of the primary cohort by *AR* status

*n* (%)	Enzalutamide chemotherapy- naïve (*n*=35)		Abiraterone chemotherapy- naive^a^ (*n*=38)		Enzalutamide post-docetaxel (*n*=27)		Abiraterone post-docetaxel (*n*=71)		
	*AR* normal 29 (83)	*AR* gain 6 (17)	*AR* normal 34 (89)	*AR* gain 4 (11)	*AR* normal 20 (74)	*AR* gain 7 (26)	*AR* normal 37 (52)	*AR* gain 26 (37)	*AR* mutant 8 (11)
Age, years Median (range)	73	71.5	75	75	78	81	75	73	77
	(63–91)	(63–81)	(56–87)	(66–86)	(59–87)	(65–85)	(41–82)	(41–91)	(63–86)
Serum PSA, mg/l Median (range)	28	110	15	313	23	252	56	142	144
	(2–1555)	(32–298)	(1–191)	(126–797)	(2–1899)	(11–893)	(1–3211)	(2–3150)	(1–803)
Serum LDH, U/l Median (range)	164	169	154	219	154	201	172	222	250
	(80–915)	(137–253)	(77–253)	(134–312)	(78–234)	(167–245)	(106–417)	(135–968)	(157–650)
Serum ALP, U/l Median (range)	76	65	92	175	90	241	93.5	96	119
	(44–531)	(55–188)	(51–426)	(102–255)	(55–531)	(87–890)	(61–934)	(36–1040)	(39–891)
Prior cabazitaxel, *n* (%)	–	–	–	–	2 (10)	1 (14)	0 (0)	3 (11)	1 (12.5)
Sites of metastases, *n* (%)									
≤5 bone metastases^b^	6 (21),0 (0)	1 (17), 0 (0)	13 (38), 0 (0)	1(25), 0 (0)	5 (40), 0 (0)	3 (43), 0 (0)	12 (32), 3 (8)	8 (31), 2 (8)	3 (37.5), 1 (12.5)
>5 bone metastases^b^	4 (14),0 (0)	2 (33), 0 (0)	14 (41), 2 (6)	3 (75), 0 (0)	12 (60), 2 (10)	4 (57), 1 (14)	17 (46), 2 (5)	17 (65), 4 (15)	5 (62.5), 1 (12.5)
Lymph node, no bone^b^ metastases	4 (14), 0 (0)	0 (0), 0 (0)	5 (15), 1 (3)	0 (0), 0 (0)	1 (5), 0 (0)	0 (0), 0 (0)	6 (16), 1 (3)	1 (4), 1 (4)	0 (0), 0 (0)
Plasma dsDNA concentration, ng/ml Median (range)	17	15	19	39	27	40	24	65	32
	(6–577)	(11–27)	(6–103)	(29–134)	(7–190)	(9–121)	(4–783)	(7–2566)	(11–550)
Time of follow-up, months Median (range)	27.8 (5.2–33.0)		18.5 (0.9–28.5)		26.1 (0.8–39.9)		44.5 (1.1–68.0)		

a No *AR* (p.L702H or p.T878A) mutation detected.

b -, visceral metastases, *n*(%).

*AR*, androgen receptor; *n*, number; PSA, prostate-specific antigen; LDH, lactate dehydrogenase; ALP, alkaline phosphatase; dsDNA, double-stranded DNA.

### Analytic testing of multiplex ddPCR for determination of plasma *AR* status

We used an optimized multiplex *AR* CN ddPCR assay on 2–4 ng DNA from all pre-treatment samples and an additional 42 samples collected after disease progression. On a further 2–4 ng DNA, we tested for *AR* mutations. From patients in the primary cohort with ddPCR data, we had NGS data available from our previous publication [8] for 86 samples and we carried out NGS on an additional 75 (samples described in supplementary Table S1, available at *Annals of Oncology* online). We observed a strong agreement between NGS and ddPCR for CN quantitation (*n *=* *161, Bland–Altman test: mean difference, −0.02, 95% CI Limits of agreement, −2.45 to 2.41) (supplementary Figure S1A and Table S2, available at *Annals of Oncology* online). Estimation of *AR* mutation allelic frequency by ddPCR also displayed strong agreement with NGS (*n *=* *60, Bland–Altman test: mean difference −0.001, 95% CI limits of agreement, −0.015 to 0.016) with no cases of mutations detected by one approach but not the other (supplementary Figure S1B, available at *Annals of Oncology* online).

### Plasma *AR* status in the primary cohort

In our primary cohort, eight post-docetaxel (but no chemotherapy-naïve) abiraterone patients were *AR* point mutation positive before treatment (Table 1). We planned to analyze these separately for associations with outcome. All four patients with a 2105T>A (p.L702H) mutation had received at least 6 months of treatment with prednisone. We did not detect a 2629T>C (p.F877L) *AR* point mutation before treatment or in an additional 26 samples collected after progression on enzalutamide. Using maximum likelihood ratio as correlative statistics combined with boot-strapping, we identified an *AR* CN cut-point of 2.01 [interquartile range (IQR), 1.82–2.77 copies] for splitting patients into two distinct prognostic groups (supplementary Figure S2, available at *Annals of Oncology* online). Use of this cut-off was also supported by 95.5% concordance between NGS and ddPCR for classifying *AR* CN status (supplementary Table S2, available at *Annals of Oncology* online). Overall, 10 (14%) chemotherapy-naïve and 33 (34%) docetaxel-treated patients had *AR* gain (Table 1).

### Plasma *AR* associates with worse outcome in the primary cohort

**Figure 1.**
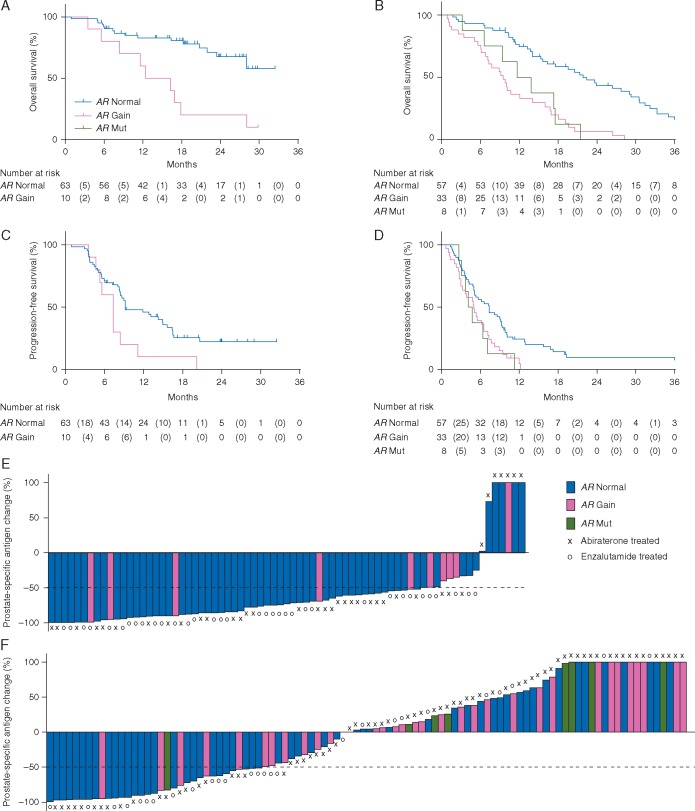
Association of plasma *AR* status with outcome in the primary cohort. Overall and progression-free survival for *AR* copy number normal, gain and mutated (Mut, p.L702H or p.T878A) chemotherapy-naïve (A, C) and post-docetaxel (B, D) castration-resistant prostate cancer patients treated with enzalutamide or abiraterone. Waterfall plots showing prostate-specific antigen (PSA) declines by *AR* copy number normal, gain and mutated (Mut, p.L702H or p.T878A) chemotherapy-naïve (E) and post-docetaxel (F) castration-resistant prostate cancer patients treated with abiraterone or enzalutamide (as marked). Bars clipped at maximum 100%.

There was a significant association for *AR* gain and OS in both chemotherapy-naïve (median 12.40 months versus not reached; HR 3.98; 95% CI 1.74–9.10; *P* < 0.001) (Figure 1A), and post-docetaxel patients (median 9.51 versus 21.80 months; HR 3.81; 95% CI 2.28–6.37; *P *< 0.001) (Figure 1B). For *AR* mutants in abiraterone-treated, post-docetaxel patients, a significant association with worse survival was also seen (median 4.06 months; HR 3.26; 95% CI 1.47–not reached; *P* = 0.004) (Figure 1B). We also observed a significant association between PFS and *AR* gain for chemotherapy-naïve patients treated with enzalutamide or abiraterone (median 7.30 versus 9.20 months; HR 2.18; 95% CI 1.08–4.39; *P* = 0.03) (Figure 1C) and for post-docetaxel patients (median 5.00 versus 7.36 months; HR 1.95; 95% CI 1.23–3.11; *P* = 0.01) (Figure 1D). A trend was seen for *AR* mutants to have worse PFS (median 4.10 months; HR 2.10; 95% CI 0.98–4.51; *P* = 0.057) (Figure 1D). Interactions between *AR* CN and treatment (abiraterone versus enzalutamide) (*P* = 0.41 for OS and *P* = 0.11 for PFS) or chemotherapy status (*P *= 0.83 for OS, *P* = 0.99 for PFS) examined in the Cox models were not significant. We also evaluated the association of *AR* status with the rate of PSA decline in the chemotherapy-naïve and post-docetaxel groups. Chemotherapy-naïve patients with *AR* gain were 4.7 times less likely to have a ≥50% decline in PSA (95% CI 1.17–19.17; *P* = 0.035) (Figure 1E). Plasma *AR* gain chemotherapy-treated patients were 5.0 times less likely to have a ≥50% decline in PSA (95% CI 1.70–14.91; *P* = 0.003) (Figure 1F). For the eight *AR* mutant patients, a trend for a lower rate of ≥50% PSA decline was seen (odds ratio (OR), 6.3; 95% CI 0.72–54.59; *P* = 0.12) (Figure 1F).

### Plasma *AR* independently associates with worse outcome on multivariable analysis in the primary cohort

Plasma *AR* status and 11 baseline characteristics previously shown to be clinically relevant [25, 26] were evaluated by both univariate and multivariable analyses on the whole primary cohort. Plasma *AR* gain or mutant was most significantly associated with OS or PFS (supplementary Tables S3 and S4 available at *Annals of Oncology* online). We then carried out multivariable analysis with stepwise backwards elimination and the sole variables that remained significant were plasma *AR* status (HR 4.10; 95% CI 2.66–6.35; *P* < 0.001, and HR 4.02; 95% CI 1.87–8.66; *P* < 0.001, for *AR* CN and *AR* mutant, respectively, Table 2A) and total plasma DNA concentration for OS and plasma *AR* status (HR 2.06; 95% CI 1.36–3.12; *P* = 0.001, and HR 2.20; 95% CI 1.03–4.69; *P* = 0.041, for *AR* CN and *AR* mutant, respectively), total plasma DNA concentration and ALP levels for PFS (Table 2B).

**Table 2:** Multivariable Cox proportional hazard analysis of predictors of overall survival (A) and progression-free survival (B) for primary cohort after stepwise backwards elimination

A			
	Overall survival		
	HR	95% CI	*P*
*AR* gain (yes versus no)	4.26	2.76–6.55	<0.001
*AR* mutant (yes versus no)	3.80	1.77–8.15	0.001
dsDNA concentration (continuous variable)	1.00	1.00–1.00	<0.001


B			
	Progression-free survival		
	HR	95% CI	*P*
*AR* gain (yes versus no)	2.22	1.48–3.34	<0.001
*AR* mutant (yes versus no)	2.59	1.24–5.44	0.012
ALP (>UNL versus ≤UNL)	1.64	1.13–2.36	0.009
dsDNA concentration (continuous variable)	1.00	1.00–1.00	<0.001

HR, hazard ratio; CI, confidence interval; *AR*, androgen receptor; dsDNA, double-stranded DNA; ALP, alkaline phosphatase; UNL, upper normal limit.

### Plasma *AR* status in the PREMIERE cohort

The PREMIERE trial enrolled 98 patients in 16 sites between February 2015 and November 2015. Plasma was collected at study entry before starting enzalutamide from 94 patients who had a median follow-up of 10.6 months. Patient characteristics by plasma *AR* status are described in Table 3A.

**Table 3:** PREMIERE cohort

(A) Baseline characteristics of patients according to *AR* status			
*n* (%)	*AR* normal 83 (88)	*AR* gain 11 (12)	
Age, years	77	80	
Median (range)	(57–95)	(60–88)	
PSA, mg/l	24	59	
Median (range)	(3–4319)	(2–254)	
Prior bicalutamide at CRPC, *n* (%)	69 (83)	9 (82)	
Sites of metastases, *n* (%), visceral metastases, *n* (%)			
≤5 bone metastases	57 (69), 10 (12)	8 (73), 1 (9)	
>5 bone metastases	12 (15), 1 (1)	1 (9), 0 (0)	
Lymph node, no bone metastases	12 (15), 2 (2)	1 (9), 0 (0)	
dsDNA concentration, ng/ml	19.4	23.1	
Median (range)	(0.5–134.7)	(4.4–1584.9)	
CTC detection, *n* (%)			
Yes	28 (34)	7 (64)	
No	55 (66)	4 (36)	
Time of follow-up, months	10.8		
Median (range)	(2.8–16.7)		


(B) Multivariable Cox proportional hazard analysis of predictors of PSA progression-free survival						
	sPFS			rPFS		
	HR	95% CI	*P*	HR	95% CI	*P*
*AR* gain (yes versus no)	4.32	1.90–9.85	<0.001	5.63	2.15–14.74	<0.001
dsDNA concentration (continuous variable)	1.00	1.00–1.00	0.240	1.00	1.00–1.00	0.853
CTC detection (AdnaTest^®^) (yes versus no)	3.18	1.63–6.20	0.001	5.74	2.08–15.90	0.001

*AR*, androgen receptor; *n*, number; PSA, prostate-specific antigen; CRPC, castration-resistant prostate cancer; dsDNA, double-stranded DNA; CTC, circulating tumor cell; sPFS, PSA progression free survival; rPFS, radiographic progression free survival; HR, hazard ratio.

### Plasma *AR* associates with worse outcome in the PREMIERE cohort

**Figure 2.**
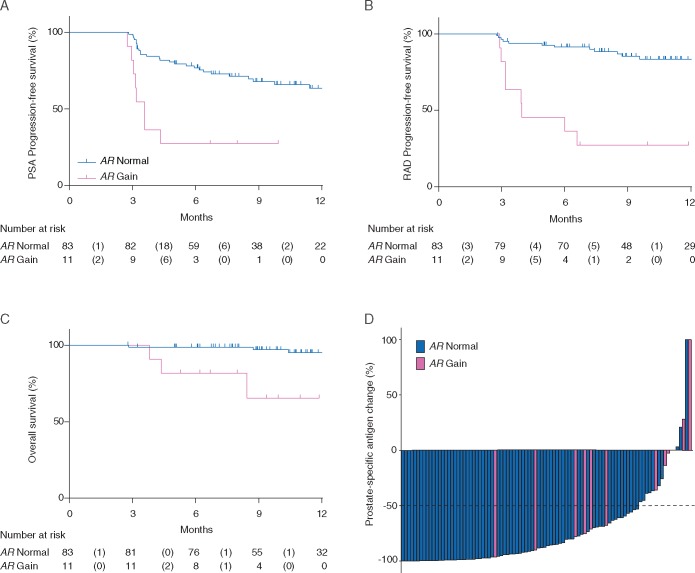
Association of plasma *AR* status with outcome in PREMIERE cohort. Prostate-specific antigen (PSA) progression-free survival (A), radiographic (RAD) progression-free survival (B) and overall survival (C) for *AR* copy number normal versus *AR* gain patients. Waterfall plot (D) showing the magnitude of PSA decline by *AR* status. Bars clipped at maximum 100%.

Similar to our primary cohort pre-chemotherapy population, we observed *AR* gain in 11 (12%) patients. CTCs were detected in 35 patients (37%). *AR* gain was detected in seven (20%) CTC-positive and four (7%) CTC-negative patients (Table 3A). Plasma *AR* gain was significantly associated with shorter sPFS (median 3.60 versus 15.5 months; HR 4.33; 95% CI 1.94–9.68; *P* < 0.001) (Figure 2A), rPFS (median 3.90 months versus not reached; HR 8.06; 95% CI 3.26–19.93; *P* < 0.001) (Figure 2B) and OS (medians not reached; HR 11.08; 95% CI 2.16–56.95; *P* = 0.004) (Figure 2C) (supplementary Table S5, available at *Annals of Oncology* online). Patients with *AR* gain were less likely to have a ≥50% decline in PSA (OR 4.93; 95% CI 1.30–18.75; *P* = 0.025) (Figure 2D).

### Plasma *AR* independently associates with worse outcome on multivariable analysis in the PREMIERE cohort

On multivariable analysis, the association of *AR* gain with the primary endpoint of sPFS was independent of plasma DNA concentration and the detection of CTCs (HR 4.32; 95% CI 1.90–9.85; *P* < 0.001) (Table 3B). *AR* gain was also independently associated on multivariable analysis with rPFS (HR 5.63; 95% CI 2.15–14.74; *P* < 0.001) (Table 3B).

## Discussion

Several treatments are available for metastatic CRPC but to date, no approved biomarker to personalize therapy. Our analyses of plasma from 265 patients collected in three prospective biomarker protocols show that the detection of *AR* CN gain before starting enzalutamide or abiraterone is associated with decreased OS and PFS regardless of prior chemotherapy status. We excluded samples from patients that had prior treatment with enzalutamide or abiraterone, given response rates and duration of benefit are very different when used sequentially [27]. Our previous study [8] suggests a similar association between plasma *AR* and resistance in patients previously treated with enzalutamide or abiraterone and this requires further investigation in future studies.

We did not detect *AR* mutations (p.T878A or p.L702H) in chemotherapy-naïve patients. Our assay detects point mutations present in at least 2% of plasma DNA. Greater sensitivity is obtained with higher input DNA [28] although the clinical relevance of rarer mutations is uncertain. By using a multiplex ddPCR with four carefully selected reference genes, we have designed a robust assay that does not over-call gain due to loss in regions involving the reference gene. Our model for estimating the likelihood of the *AR* CN cut-off that best predicts associations with outcome was built with 171 patients. We plan to perform a meta-analysis of multiple trials when the data on *AR* CN acquired from different institutions and trials exceeds 1000 patients.

Detection of AR splice variants in CTCs is also associated with shorter PFS and OS with enzalutamide or abiraterone [19, 29]. *AR* CN is higher in the population with detectable CTCs although *AR* gain can also be observed in CTC-negative patients, accounting for one third of *AR* gained in the PREMIERE cohort. The overlap between AR-V7 positive and plasma *AR* gained patients and a comparison of the two tests in prospective trials is warranted to develop the best biomarker strategy for identifying resistant patients. Testing plasma *AR* status by ddPCR is affordable and can be widely implemented in clinical laboratories but does not control for plasma DNA tumor content [7, 8] that may introduce a bias. Nonetheless, multivariable analyses confirm that plasma *AR* by ddPCR provides information on the outcome of men starting enzalutamide or abiraterone that is independent of other factors previously reported to be prognostic [25, 26, 30]. In keeping with higher response rates to AR targeting in chemotherapy-naïve patients, the prevalence of plasma *AR* aberrations is 10%–15% in this setting compared with 30%–40% post-docetaxel. As our study is single arm, the associations we report are prognostic although the association with PSA decline rate suggests plasma *AR* CN could identify patients resistant to enzalutamide or abiraterone. The aims of our study were defined after sample collection and therefore larger studies with a pre-specified primary objective of defining the association with outcome by plasma *AR* status could provide further supportive evidence for the role of *AR* CN as a biomarker in CRPC. For level one evidence to change clinical practice, our findings require confirmation in prospective trials where plasma *AR* CN defines treatment selection.

## Supplementary Material

Supplementary Data

## Appendix

### PREMIERE Collaborators

T. Alonso, Servicio de Oncología Médica, Hospital Ramón y Cajal, Madrid, Spain; J. Tudela, Servicio de Anatomía Patológica, Hospital Morales Meseguer, Murcia, Spain; A. Martinez, Biobanco de la Región de Murcia, IMIB Nodo 3, Murcia, Spain.
