# Supplementary material for: Androgen receptor gene status in plasma DNA associates with worse outcome on enzalutamide or abiraterone for castration-resistant prostate cancer: a multi-institution correlative biomarker study
Source: Ann Oncol. 2017 May 3;28(7):1508–16. doi: 10.1093/annonc/mdx155 (PMC5834043; doi:10.1093/annonc/mdx155)
Supplement: Supplementary Data [file mdx155_supp.docx]

**Supplementary Online Material**

**Appendix S1.**

1. Eligibility criteria of Primary cohort…………………………………………………....……………...…..…....…2

2. Eligibility criteria of PREMIERE cohort…………………………………………………....……………...…..….3

**Appendix S2**. Detection of *AR* aberrations by digital droplet PCR in plasma samples…...………...…..........5

**Appendix S3.** Statistical Analysis………….………………………………………………………………………..6

**Supplementary Figures**

**Figure S1.** Evaluation of ddPCR copy number (CN) and mutation assay performance ………...………..7

**Figure S2.** Selection of cut-off for *AR* CN gain by ddPCR……………...……………….....................….….8

**Supplementary Tables**

**Table S1.** Samples performed by both NGS and ddPCR……...……….......................….…………...…….9

**Table S2.** Concordance of *AR* CN estimation between NGS and ddPCR………………………….….….10

**Table S3.** Univariate analysis in the primary cohort……………...…..............………………...…………...11

**Table S4.** Multivariable Cox Proportional Hazard analysis of predictors of overall survival and progression-free survival for primary cohort……………...…..............………………...…………...............12

**Table S5.** Univariate analysis in PREMIERE. ………………………………………………….….……..…..13

**Supplementary References**………….……………..……………………..……………………...……………....14

**Appendix S1**

**1. Eligibility Criteria of Primary cohort**

**Inclusion Criteria**

1. Patients must have histologically-confirmed adenocarcinoma of prostate without neuroendocrine differentiation or small cell histology.

2. Patients have progressive disease despite “castration levels” of serum testosterone (<50 ng/dL) (≤1.73 nmol/L), and ongoing LHRH analogue treatment or prior surgical castration.

3. Progression as defined by at least two of the following: a rise in PSA, worsening symptoms, or radiological progression, namely, progression in soft tissue lesions measured by computed tomography imaging according to the modified Response Evaluation Criteria in Solid Tumors (RECIST) or progression on bone scanning according to criteria adapted from the Prostate Cancer Working Group (PCWG2) criteria.

4. Patients have not received radiotherapy, chemotherapy, or immunotherapy at least 30 days prior to the treatment.

5. Male, aged ≥18 years.

6. Life expectancy of greater than three months.

7. Eastern Cooperative Oncology Group (ECOG) Performance Status ≤2.

8. Able to swallow the study drug whole as a tablet.

9. Willing to use a method of birth control with adequate barrier protection.

10. Patients must have normal organ and marrow function as defined below:

a. leukocytes >3,000/mL

b. absolute neutrophil count >1,500/mL

c. platelets >100,000/mL

d. total bilirubin within normal institutional limits

e. AST(SGOT)/ALT(SGPT) <2.5 X institutional upper limit of normal

f. creatinine within normal institutional limits

11. No evidence (within five years) of prior malignancies (except successfully treated basal cell or squamous cell carcinoma of the skin).

12. Participant is willing and able to give informed consent for participation in the study.

**Exclusion Criteria**

1. Patients who have had previous therapy with abiraterone and/or enzalutamide.

2. Concurrent use of other anticancer agents or treatments, with the following exceptions:

a. LHRH agonists or antagonists

b. denosumab or bisphosphonate (e.g., zoledronic acid).

3. Uncontrolled intercurrent illness including, but not limited to, ongoing or active infection, symptomatic congestive heart failure, unstable angina pectoris, cardiac arrhythmia, or psychiatric illness/social situations that would limit compliance with study requirements.

4. History of seizures or any disease that could predispose to seizure, including history of lost of consciousness or transient stroke in the last 12 months before inclusion (day 1).

5. Have a history of gastrointestinal disorders that may interfere with the absorption of the study agents.

6. Have a pre-existing condition that warrants long-term corticosteroid use in excess of study dose.

7. Have known allergies, hypersensitivity or intolerance to abiraterone acetate, prednisone, enzalutamide, or their excipients.

8. Other primary tumor (other than CRPC) including hematological malignancy present within the last five years (except non-melanoma skin cancer or low-grade superficial bladder cancer).

**2. Eligibility Criteria of PREMIERE cohort**

**Inclusion criteria.**

1. Age ≥18 years old.
2. Histologically or cytologically confirmed of prostate adenocarcinoma without neuroendocrine differentiation or small cell characteristics.
3. Ongoing androgen deprivation with GnRH analog or bilateral orchiectomy.
4. Testosterone serum levels ≤1.73 nmol/L or 50 ng/dL at the screening visit.
5. Patients receiving bisfosfonate therapy must have been on stable doses for at least four weeks before study entry.
6. Progressive disease at study entry, defined by one or more of the three following criteria while the patient was on androgen deprivation therapy:
7. PSA progression defined by a minimum of two rising PSA values with an interval of ≥one week between each determination. Patients that have received anti-androgen must be in progression upon anti-androgen withdrawal at least four weeks for flutamide and six weeks since the last dose of bicalutamide or nilutamide. PSA value should be ≥2 µg/L (2 ng/mL).
8. Progression in soft tissue according to RECIST 1.1
9. Bone progression defined by the PCWG2 criteria, at least two new more lesions in the bone scan.
10. Metastatic disease documented by bone lesions in bone scan or by measurable soft tissue lesions by CT or MRI. Patients whose disease was limited to lymph nodes were required to have a lesion with a minor diameter of 2.5 cm.
11. No prior cytotoxic chemotherapy for prostate cancer.
12. Patients without previous abiraterone acetate treatment.
13. Asymptomatic or minimally symptomatic disease from prostate cancer (i.e., the score on Brief Pain Inventory question Short form question #3 must be <4).
14. Eastern Cooperative Oncology Group (ECOG) performance status of 0-1.
15. Estimated life expectancy of ≥ six months.
16. Patient able to swallow the study drug and to follow-up the study requirements.
17. Informed consent for the biomarker study: *TMPRSS2-ETS* rearrangement and the obtained samples.

**Exclusion criteria.**

1. Comorbidity, infection or severe concurrent disease, in the judgment of the investigator, that makes the patient not suitable for inclusion in the study.
2. Known or suspicion of brain or leptomeningeal disease.
3. History of another malignancy within the previous 5 years other than cured non-melanoma skin cancer.
4. Hematological count at screening selection:
5. Absolute neutrophil count <1,500/µL
6. Platelet count <100,000/µL
7. Haemoglobin <5.6 mmol/L (9 g/dL)
8. Liver function at the screening visit: total bilirubin, aminotransferase (ALT) or aspartate aminotransferase (AST) >2.5 times upper normal limit.
9. Renal function at the screening visit: creatinine >177 µmol/L.
10. Albumin value <30 g/L (3 g/dL) at the screening visit.
11. History of seizures or any disease that could predispose to seizure, including history of lost of consciousness or transient stroke in the last 12 months before inclusion (day 1).
12. Clinically significant cardiovascular disease, including:
13. Myocardial infarction within six months
14. Uncontrolled angina within three months
15. Congestive heart failure New York Heart Association (NYHA) class III or IV or history of congestive heart failure class III or IV in the past, unless a screening echocardiogram or multi-gated acquisition scan performed within three months results in a left ventricular ejection fraction ≥45%.
16. History of clinically significant ventricular arrhythmias (e.g., ventricular tachycardia)
17. Heart block (Mobitz II or III without a permanent pace-maker in place.
18. Hypotension at the screening visit, as indicated by systolic blood pressure <86 mmHg)
19. Bradycardia as indicate by a heart rate of <50 beats per minute on the screening ECG.
20. Uncontrolled hypertension as indicated by systolic blood pressure >170 or diastolic blood pressure >105 rpm at the screening visit.
21. Gastrointestinal disorder affecting absorption (e.g., gastrectomy, active peptic ulcer disease within three months).
22. Major surgery within last four months of inclusion.
23. Use of opioids for pain within four weeks before screening visit.
24. Use of radiotherapy for the treatment of the primary tumor within three weeks before treatment.
25. Use of radiotherapy for the treatment of metastasis within two months before study entry.
26. Use of radium-223 or other radionuclides for the treatment of bone disseminated disease.
27. Treatment with flutamide within four weeks of enrollment.
28. Treatment with bicalutamide or nilutamide within six weeks before enrollment in the study.
29. Treatment with 5-α reductase (finasteride, dutasteride), estrogens or ciproterone acetate within four weeks of enrollment.
30. Treatment with biological therapy for prostate cancer (other than bone targeted agents and GnRH analogues) or other drugs with antitumoral activity in the four weeks before study entry.
31. History of prostate cancer progression on ketoconazole.
32. Previous use, or participation in a clinical trial, of an investigational drug that blocks androgen synthesis (e.g., abiraterone, TAK-100, TAC 683, TAK-448) or target the androgen receptor (e.g., ARN507, BMS641988).
33. Participation in a clinical trial including enzalutamide.
34. Use of an investigational drug in the four weeks of enrollment.
35. Use of herbal products that may have hormonal anti-cancer activity or that modify PSA levels, systemic steroids at a dose higher than the equivalent of 10 mg of prednisone within four weeks of enrollment.
36. Hereditary fructose intolerance.

Any condition or reason that in the opinion of the investigator interferes with the ability of the patient to participate in the trial, which places the patient at undue risk, or complicates the interpretation of safety data.

**Appendix S2. Detection of *AR* aberrations by digital droplet PCR in plasma samples**

Circulating DNA was extracted from one to two ml of plasma with the QIAamp Circulating Nucleic Acid Kit (Qiagen). Total extracted plasma DNA was quantified with the Quant-iT high sensitivity PicoGreen double-stranded DNA Assay Kit (Invitrogen). DdPCR was performed on a QX200 ddPCR system (Bio-Rad). Copy number (CN) assays were performed for *AR* (Hs04121925_cn, FAM) and centromeric chromosome X gene *ZXDB* (Hs02220689_cn, FAM, Life Technologies) with *NSUN3* (dHsaCP2506682, HEX, Bio-Rad), *ElF2C1* (dHsaCP1000002, HEX, Bio-Rad), and *AP3B1* (dHsaCP1000001, HEX, Bio-Rad) as reference genes. We developed multiplex assays by varying the concentration of the fluorescent probes to differentiate droplets positive for respective genes on the basis of fluorescence intensity [1-3].

Rare mutation detection assays were performed for the *AR* mutations 2105T>A (p.L702H), 2632A>G (p.T878A), and 2629T>C (p.F876L) using a custom-made single nucleotide polymorphism (SNP) genotyping assay (Life Technologies), the SNP genotyping assay rs137852581 (Life Technologies), and the SNP genotyping assay rs137852578 (Life Technologies), respectively.

PCR reactions were prepared with 1-2 ng DNA, 10ul 2xSupermix and a total volume of primer probe assays of 2ul in a total volume of 20ul. PCR reactions were partitioned into ~20,000 droplets per sample with an Automated Droplet generator (Bio-Rad). Emulsified PCR reactions were run on a Mastercycler Nexus GSX1 (Eppendorf). For mutation assays, ddPCR conditions were optimized with a temperature gradient to identify the optimal annealing/extension temperature using wild-type DNA spiked with a synthetic oligonucleotide containing the mutation of interest. We selected the optimal temperature for incubation on the Mastercycler Nexus GSX1. Samples were incubated at 99°C for 10 min followed by 40 cycles of 95°C for 15 sec, 60°C for 60 sec, followed by 10 min incubation at 98°C for the *AR* copy number multiplex assay. For *AR* mutation detection, samples were incubated at 99°C for 10 min followed by 40 cycles of 95°C for 15 sec, 56-61°C for 60 sec, followed by 10 min incubation at 98°C.

Samples were read on a Bio-Rad QX200 droplet reader using QuantaSoft v1.3.2.0 software for *AR* CN analysis and mutation detection. At least two negative control wells with no DNA were included in every run. An oligo carrying the mutation of interest was used as a positive control for mutation assays. In addition, two wells with DNA from a germ line sample, characterized by the complete absence of mutation and normal *AR* CN status, were also included. Positive and negative clusters were gated using the FAM and VIC/HEX thresholds based on the amplitude of positive and negative controls that were ran concomitantly with each assay. Poisson distribution was used to estimate the average number of copies per reaction microliters. CN ratios of *AR* and reference genes and mutant vs wild-type were calculated for each sample to determine *AR* CN and the mutation allele fraction respectively as described previously [4].

**Appendix S3. Statistical analysis**

Using NGS we previously used *AR* amplicon variance in healthy volunteer plasma to set a cut-off of 1.91 for calling a patient CN gain. We do not observe variance with ddPCR and could therefore theoretically choose any cut-off >1. We performed a systematic search over all observed values of *AR* CN to identify the *AR* CN, which optimally splits the patients into two groups who have different prognosis of overall survival as we had hypothesized that *AR* gained patients have higher hazard rates than *AR n*ormal patients. *AR* mutant patients were excluded for this research. We used log-likelihood as correlative measure in a multivariable Cox proportional hazard model which included *AR* CN and serum lactate dehydrogenase as the second variable and was stratified by chemotherapy status of the patients It has been shown that multivariable approach increases the accuracy of the cutpoint [5]. We used bootstrapping with replacement technique and iterated the search for the optimal cutpoint 30,000 times to estimate the measures of dispersion of the cutpoint. The search for cutpoint and the bootstrapping were performed using an in-house developed R script (supplementary Figure S2, available at *Annals of Oncology* online).

The association of *AR* status with progression-free survival (PFS) and overall survival (OS) was evaluated using univariable Cox regression. Survivor function of Time-to-event outcomes were also estimated using the Kaplan-Meier method. Differences between survivor functions of patients with *AR* CN gain *vs* *AR* CN normal (and *AR* mutant vs *AR* no mutant in docetaxel-treated patient group) were evaluated using the log-rank test. The association of *AR* status with time-to event outcomes was evaluated and hazard ratios (HRs) estimated from univariable and multivariable Cox proportional hazards regression methods (Figure 1A-C and Figure 2A-C).

Best PSA responses were depicted using standard waterfall plots; odds ratios (ORs) and the corresponding 95% confidence interval (CI) of PSA response were determined using a 2x2 contingency table and the Woolf logit method. Statistical significance was determined using Fisher’s exact test (Figure 1E-F and Figure 2D).

The pre-treatment predictors evaluated for the multivariable Cox proportional hazards models included *AR* CN (gain *vs* normal), *AR* mutant (yes *vs* no), lactate dehydrogenase levels [>upper normal limit (UNL) *vs* ≤UNL], presence of liver metastases (yes *vs* no), presence of bone metastases (≤5 *vs* >5), neutrophil-to-limphocyte ratio (>3 *vs* <3), alkaline phosphatase levels (>UNL *vs* ≤UNL), hemoglobin levels (≥UNL *vs* <UNL), albumin levels (>UNL *vs* ≤UNL), previous chemotherapy (yes *vs* no), dsDNA concentration (continuous variable), PSA levels (continuous variable), and patient age (continuous variable) (supplementary Table S4 and Table S5B, available at *Annals of Oncology* online). The final multivariable analyses were assessed using a proportional hazard model after stepwise backwards elimination by Akaike information criterion (Table 2).

**Supplementary Figures**

**A**

**
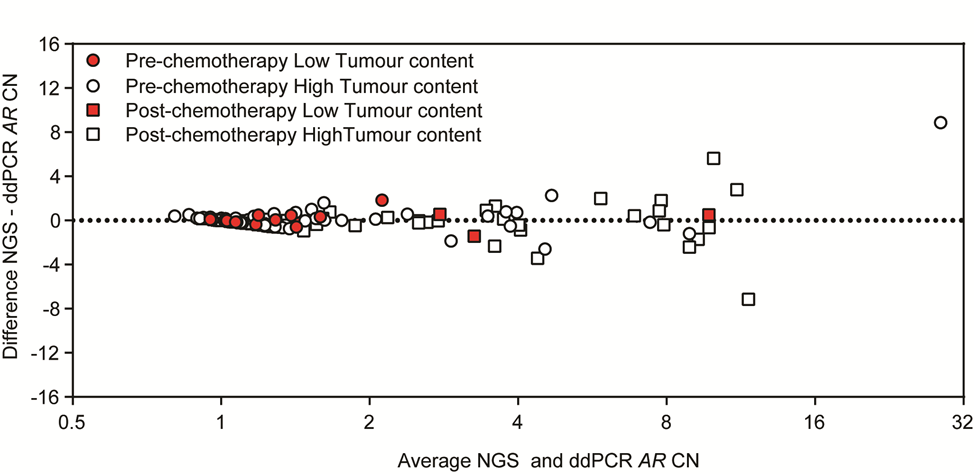
**

**B**

**
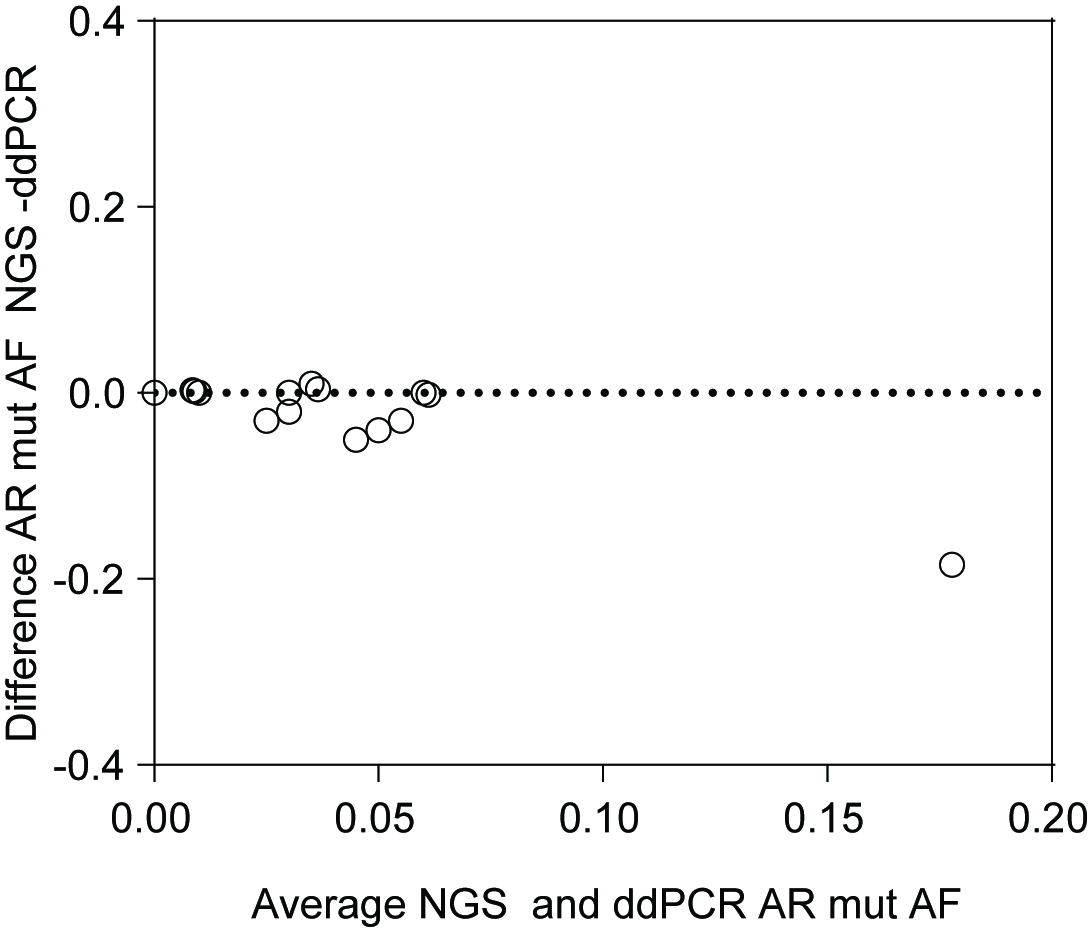
**

**Figure S1. Evaluation of ddPCR copy number (CN) and mutation assay performance.** Bland-Altman plot showing agreement of ddPCR and NGS *AR* copy number assessment, low tumor content samples had a tumor content fraction below 0.075 (A). Bland-Altman plot showing agreement of ddPCR and NGS *AR* mutation frequencies (B).

**A**

­
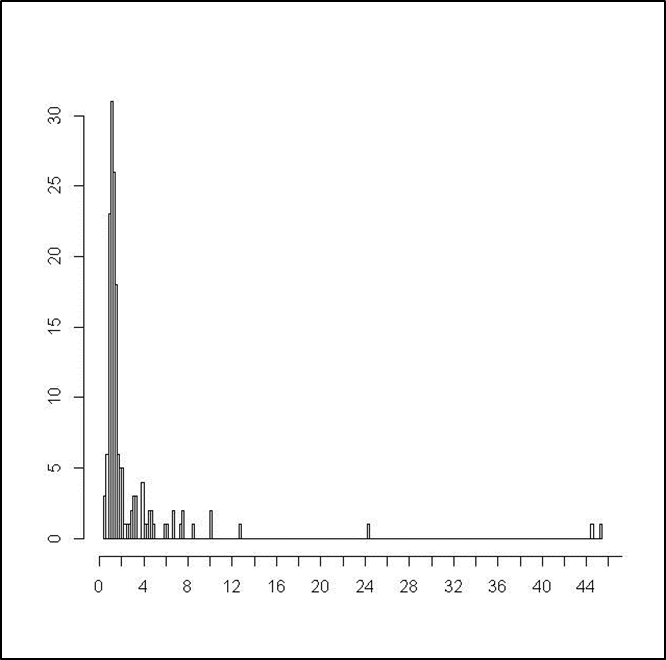


**B**


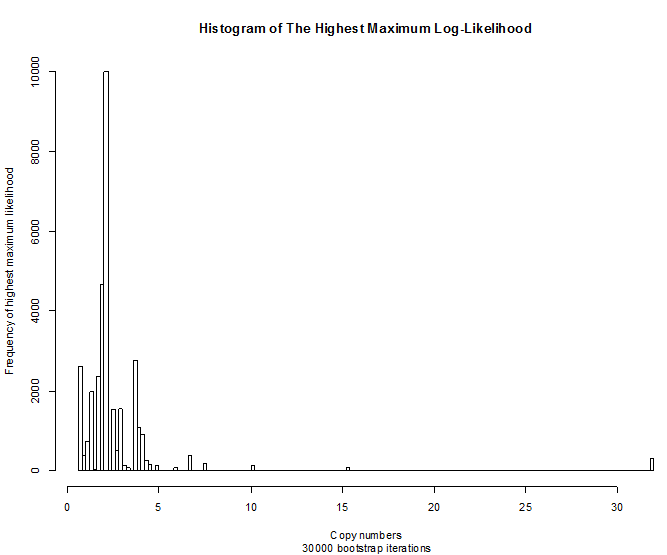


**Figure S2. Selection of cut-off for *AR* CN gain by ddPCR.** Range of *AR* CN across primary cohort (A), Cut-off analysis with maximum log-likelihood as the correlative statistic of the multivariable Cox proportional hazard model and boot-strapping with 30,000 iterations to provide the cut-off point dispersion (B).

**­**

**Supplementary Tables**

**Table S1. Samples analysed by both NGS and ddPCR**

|  |  | Pretreatment (*n*) | Progression (*n*) |
| --- | --- | --- | --- |
| NGS data included in previously published cohort [6] | Chemotherapy-naive | 8 | 1 |
|  | Post-docetaxel | 58 | 19 |
| NGS data not in previously published cohort | Chemotherapy-naive | 53 | 22 |
|  | Post-docetaxel | 0 | 0 |
|  | Total | 119 | 42 |

*Abbreviations.* ddPCR, droplet digital PCR; *n,* number; NGS, next generation sequencing.

**Table S2. Agreement of *AR* CN gain call by ddPCR vs NGS**

| **Chemotherapy-naive**  **Cut off 2.01** | ***AR* Normal NGS** | ***AR* Gain NGS** | **NGS TC <0.075** |
| --- | --- | --- | --- |
| *AR* Normal ddPCR | 57 | 3 | 12 |
| *AR* Gain ddPCR | 1 | 10 | 0 |
| **Post-docetaxel**  **Cut off 2.01** | ***AR* Normal NGS** | ***AR* Gain NGS** | **NGS TC<0.075** |
| *AR* Normal ddPCR | 37 | 1 | 12 |
| *AR* Gain ddPCR | 1 | 23 | 3 |

*Abbreviations. AR*, androgen receptor; ddPCR, digital droplet PCR; NGS, next generation sequencing; TC,

tumor content.

**Table S3. Univariate analysis in the primary cohort**

|  | **Overall Survival** | | | **Progression-free Survival** | | |
| --- | --- | --- | --- | --- | --- | --- |
|  | **HR** | **CI 95%** | ***p*** | **HR** | **CI 95%** | ***p*** |
| **AR gain**  (yes vs no) | 4.07 | 2.68-6.20 | < 0.001 | 2.33 | 1.61-3.36 | < 0.001 |
| **AR mutant**  (yes vs no) | 4.81 | 2.02-11.44 | < 0.001 | 2.86 | 1.24-6.59 | 0.014 |
| **Previous chemotherapy**  (yes vs no) | 2.38 | 1.51-3.75 | < 0.001 | 1.92 | 1.36-2.71 | < 0.001 |
| **Pretreatment dsDNA concentration**  (continuous variable) | 1.00 | 1.00-1.00 | < 0.001 | 1.00 | 1.00-1.00 | < 0.001 |
| **Pretreatment LDH**  (>UNL vs ≤UNL) | 2.21 | 1.50-3.24 | < 0.001 | 1.91 | 1.35-2.68 | < 0.001 |
| **Liver metastases**  (yes vs no) | 2.61 | 1.35-5.02 | 0.004 | 1.61 | 0.84-3.08 | 0.147 |
| **Bone metastases**  (>5 vs ≤5) | 1.68 | 1.15-2.46 | 0.007 | 1.49 | 1.07-2.07 | 0.017 |
| **NLR**  (>3 vs <3) | 1.67 | 1.13-2.46 | 0.010 | 1.34 | 0.96-1.87 | 0.080 |
| **ALP**  (>UNL vs ≤UNL) | 2.00 | 1.36-2.93 | 0.010 | 2.09 | 1.48-2.94 | < 0.001 |
| **Hb**  (<UNL vs ≥UNL) | 1.80 | 1.20-2.69 | 0.004 | 1.50 | 1.03-2.18 | 0.031 |
| **Albumin**  (<UNL vs ≥UNL) | 1.41 | 0.92-2.15 | 0.110 | 1.32 | 0.93-1.87 | 0.120 |
| **PSA**  (continuous variable) | 1.00 | 1.00-1.00 | 0.009 | 1.00 | 1.00-1.00 | 0.002 |
| **Age**  (continuous variable) | 0.98 | 0.95-1.00 | 0.054 | 0.98 | 0.96-1.00 | 0.104 |

*Abbreviations.* ALP alkaline phosphatase; *AR*, androgen receptor; CI, confidence interval; dsDNA, double-stranded DNA; Hb, hemoglobin; HR, hazard ratio; LDH, lactate dehydrogenase; NLR, neutrophil to lymphocyte ratio; PSA, prostate specific antigen; UNL, upper normal limit.

**Table S4. Multivariable Cox Proportional Hazard Analysis of predictors of overall survival and progression-free survival for primary cohort**

|  | **Overall Survival** | | | **Progression-free Survival** | | |
| --- | --- | --- | --- | --- | --- | --- |
|  | **HR** | **CI 95%** | ***p*** | **HR** | **CI 95%** | ***p*** |
| **AR gain**  (yes vs no) | 3.81 | 2.37-6.12 | < 0.001 | 2.05 | 1.31-3.19 | 0.002 |
| **AR mutant**  (yes vs no) | 3.12 | 1.32-7.40 | 0.010 | 2.23 | 0.98-5.08 | 0.056 |
| **Previous chemotherapy**  (yes vs no) | 1.27 | 0.72-2.23 | 0.407 | 1.39 | 0.89-2.17 | 0.147 |
| **Pretreatment dsDNA concentration**  (continuous variable) | 1.00 | 1.00-1.00 | 0.010 | 1.00 | 1.00-1.00 | < 0.001 |
| **Pretreatment LDH**  (>UNL vs ≤UNL) | 1.31 | 0.81-2.11 | 0.273 | 1.21 | 0.79-1.87 | 0.379 |
| **Liver metastases**  (yes vs no) | 1.49 | 0.69-3.21 | 0.312 | 0.76 | 0.34-1.68 | 0.493 |
| **Bone metastases**  (>5 vs ≤5) | 1.35 | 0.87-2.11 | 0.184 | 1.22 | 0.83-1.79 | 0.304 |
| **NLR**  (>3 vs <3) | 1.37 | 0.89-2.11 | 0.156 | 1.06 | 0.73-1.54 | 0.759 |
| **ALP**  (>UNL vs ≤UNL) | 1.32 | 0.85-2.05 | 0.222 | 1.43 | 0.95-2.14 | 0.086 |
| **Hb**  (<UNL vs ≥UNL) | 0.91 | 0.55-1.50 | 0.705 | 0.79 | 0.49-1.26 | 0.314 |
| **Albumin**  (<UNL vs ≥UNL) | 1.01 | 0.61-1.65 | 0.980 | 1.07 | 0.71-1.62 | 0.730 |
| **PSA**  (continuous variable) | 1.00 | 1.00-1.00 | 0.458 | 1.00 | 1.00-1.00 | 0.766 |
| **Age**  (continuous variable) | 0.99 | 0.96-1.02 | 0.386 | 0.99 | 0.96-1.01 | 0.309 |

*Abbreviations.* ALP alkaline phosphatase; *AR*, androgen receptor; CI, confidence interval; dsDNA, double-stranded DNA; Hb, hemoglobin; HR, hazard ratio; LDH, lactate dehydrogenase; NLR, neutrophil to lymphocyte ratio; PSA, prostate specific antigen; UNL, upper normal limit.

**Table S5. Univariate analysis in PREMIERE.** Biochemical PFS (A) and radiographic PFS (B)

**A**

|  | **sPFS** | | |
| --- | --- | --- | --- |
|  | **HR** | **CI 95%** | ***p*** |
| ***AR* gain**  (yes vs no) | 4.33 | 1.94-9.68 | < 0.001 |
| **Pretreatment dsDNA concentration**  (continuous variable) | 1.00 | 1.00-1.00 | 0.779 |
| **CTCs (AdnaTest®)**  (detected vs not detected) | 3.40 | 1.76-6.56 | < 0.001 |

**B**

|  | **rPFS** | | |
| --- | --- | --- | --- |
|  | **HR** | **CI 95%** | ***p*** |
| ***AR* gain**  (yes vs no) | 8.06 | 3.26-19.93 | < 0.001 |
| **Pretreatment dsDNA concentration**  (continuous variable) | 1.00 | 1.00-1.00 | 0.012 |
| **CTCs (AdnaTest®)**  (detected vs not detected) | 7.09 | 2.61-19.25 | < 0.001 |

*Abbreviations. AR*, androgen receptor; CI, confidence interval; CTC, circulating tumor cell; dsDNA,

double-stranded DNA; HR, hazard ratio; sPFS, biochemical progression-free survival; rPFS,

radiographic progression-free survival.

**Supplementary References**

1. Taly V, Pekin D, Benhaim L et al. Multiplex picodroplet digital PCR to detect KRAS mutations in circulating DNA from the plasma of colorectal cancer patients. Clin Chem 2013; 59: 1722–1731.
2. Gevensleben H, Garcia-Murillas I, Graeser MK et al. Noninvasive detection of HER2 amplification with plasma DNA digital PCR. Clin Cancer Res 2013; 19: 3276-3284.
3. Garcia-Murillas I, Schiavon G, Weigelt B et al. Mutation tracking in circulating tumor DNA predicts relapse in early breast cancer. Sci Transl Med 2015; 302: 302ra133.
4. Hindson BJ, Ness KD, Masquelier DA et al. High-throughput droplet digital PCR system for absolute quantitation of DNA copy number. Anal Chem 2011; 83: 8604-8610.
5. Mazumdar M, Smith A, Bacik J. Methods for categorizing a prognostic variable in a multivariable setting. Stat Med 2003; 22: 559-571.
6. Romanel A, Gasi Tandefelt D, Conteduca V et al. Plasma AR and abiraterone-resistant prostate cancer. Sci Transl Med 2015; 312: 312re10.
